# The role of guggulsterone on the NF-κB pathway in inflammatory bowel disease: preclinical evidence

**DOI:** 10.2144/fsoa-2022-0020

**Published:** 2022-06-27

**Authors:** SK Sujitha Priya, Ankita Jha, Rajappan Chandra Satish Kumar, Sarvesh Sabarathinam

**Affiliations:** 1 Department of Pharmacy Practice, SRM Institute of Science & Technology, Kattankulathur, Kancheepuram, 603203, Tamil Nadu, India; 2 Drug Testing Laboratory, Interdisciplinary Institute of Indian System of Medicine (IIISM), SRM Institute of Science & Technology, Kattankulathur, 603203, Tamil Nadu, India

**Keywords:** bioactive compounds, cytokines, dextran sodium sulfate, guggulsterone, inflammatory bowel disease, intestinal epithelial cells, NF-κB, preclinical studies, ulcerative colitis

## Abstract

Guggulsterone, a phytosterol in the herb *Commiphora wightii* (Arn.) Bhandari, has been used in the treatment of diseases such as hyperlipidemia, arthritis and atherosclerosis. It has attracted significant research interest because of its pharmacological properties, especially its anti-inflammatory nature. It can be used for the treatment of inflammatory bowel disease (IBD), a disease characterized by chronic inflammation and damage in the gastrointestinal tract. In various preclinical studies, it inhibited NF-κB, an important pathway in the pathophysiology of IBD. This review summarizes the preclinical studies on this topic for providing more insights to the mechanism by which guggulsterone exerts its effect.

Guggul has been used for the treatment of arthritis, inflammation, obesity, cardiovascular protection, anti-ulcer, anti-ischemic, anti-epileptic and lipid metabolic issues for thousands of years. Guggulsterone isomers are the major bioactive compounds that play a major role in cholesterol metabolism [1]. This plant steroid acts as a nuclear receptor antagonist, namely the farnesoid X receptor, which is involved in bile acid and cholesterol metabolism. Guggulsterone also controls transcription factors such as NF-κB and STAT3, which are important in inflammatory and cancer processes. Guggulsterone suppresses the synthesis of proteins associated with anti-apoptotic, cellular function, proliferation and differentiation, thrombotic, metastatic and chemo-resistant activities in solid tumors. The ultimate aim of this study is to assess the NF-κB inhibition profile of the guggulsterone isomer [2].

## Overview of NF-κB

NF-κB is a group of inducible transcription factors that control a wide range of genes involved in immunological and inflammatory responses [3]. The modulation of inflammatory responses is a well-known function of NF-κB. NF-κB governs the activation, differentiation and effector function of inflammatory T cells in addition to controlling the expression of several proinflammatory genes in innate immunity. Inflammation is necessary, but dysregulated inflammation is not important; it can lead to extensive and chronic tissue damage. NF-κB plays an important role in the immune system of the intestine by modulating inflammation. Members of the NF-κB family regulate the transcriptional activity of proinflammatory cytokine promoters, cell surface receptors, transcription factors and adhesion molecules involved in intestinal inflammation. NF-κB plays an important role in regulating the expression of various genes associated with the immune function of the gut. It plays a vital role in involving many cytokine genes through transcriptional regulation, e.g., IL-1, IFN-γ, IL-2, IL-6, IL-8 and IL-12p40 (these are critical for innate and adaptive immunity). It has been studied that IL-2, IL-3 and IL-8 expressions (which play an important role in intestinal inflammation such as in inflammatory bowel disease [IBD]) are regulated by NF-κB during CD28 co-stimulation. Moreover, the role of NF-κB is not only limited to the regulation of cytokine expression but also for the regulation of a variety of genes encoding transcription factors and cell adhesion molecules [4].

## Role of pro- & anti-inflammatory cytokines in IBD

Cytokines play a vital role in the pathogenesis of various inflammatory diseases including IBD. They are secretory proteins, which are predominantly produced by T cells and macrophages. It is involved in instigating, perpetuating, maintaining as well as suppressing the inflammation and injury to the intestinal cells. IBD results from the imbalance between pro- and anti-inflammatory cytokines. Therefore, cytokines are considered as a prospective therapeutic target and are being used in clinical setup as well as for animal studies and clinical trials. Pro-inflammatory cytokines are triggered by macrophages and are involved in the upregulation of inflammation. Anti-inflammatory cytokines regulate thactivation of other pro-inflammatorye production of pro-inflammatory cytokines and reduces inflammation. Crohn's disease involves a Th-1-mediated inflammatory response which has upregulated IFN-γ, TNF-α, IL-12 and IL-18, and act as pro-inflammatory cytokines and downregulated IL-5,IL-18 and IL-21, which act as an anti-inflammatory cytokines whereas ulcerative colitis which is Th-2 mediated, all Th-2 mediated cytokines like IL-5IL-6,IL-13, IL-17 and TNF-α are upregulated and act as pro-inflammatory cytokines, whereas IL-18 is downregulated and act as an anti-inflammatory cytokine [5]. Cytokines play a complex role in IBD because both Th1 and Th2 regulate each other [6].

Several new anti-cytokine drugs have demonstrated either little or no efficacy in IBD, implying the existence of a tightly controlled cytokine network. This suggests the need to develop a potential drug therapy which acts as a multi-cytokine blocker targeting both pro- and anti-inflammatory cytokines. Governing the expression, synthesis and functional activity of other pro- and anti-inflammatory cytokines could lead to the development of a more effective, safe and less damaging therapeutic approach. NF-kB is one of the main gateways in activation of other pro-inflammatory cytokines [7]. Even though NF-kB is also necessary for optimal immune responses and cell viability, inhibition of this pathway is one of the mechanisms by which currently available anti-inflammatory drugs like steroids and aminosalicylates elicit their response [8].

## NF-κB signaling pathway in IBD

IBD is an umbrella term for two conditions: Crohn's disease and ulcerative colitis. Alhough, the exact etiology of IBD is unknown, the pathophysiology of both Crohn's disease and ulcerative colitis is linked to dysregulated cytokine production and signaling processes by epithelial cells, mucosal lymphocytes, and macrophages (i.e., disrupted mucosal immune homeostasis). The stages of IBD can be categorized based on severity; mild, moderate and severe, and on the basis of disease state; active or in remission. However, there are currently no formal validated or accepted classifications for these categorizations. In IBD, there are three key domains to consider when assessing disease severity: the disease and effect on the patient (which includes clinical symptoms), inflammatory load (severity and extent of the disease) and disease progression (structural issues and flares). The associations between these variables are not mutually exclusive.

NF-κB plays an important role in the pathogenesis of IBD, which has been backed by much evidence, in particular, inflamed colonic tissues from IBD patients have constitutive NF-κB activation. Interestingly, IL-10, sulfasalazine and other immunosuppressive medications like cyclosporin A and glucocorticoids, which are commonly used to treat patients with chronic intestinal inflammation, block NF-κB activation. The persistence of NF-κB activation in individuals with active IBD suggests that NF-κB activity modulation is a promising therapeutic target.

## Preclinical evidence of guggulsterone on NF-κB signaling pathway

One of the key bioactive chemicals found in *Commiphora* species is the guggulsterone isomer. The farnesoid X receptor, which is involved in the conversion of cholesterol to bile acids, is antagonized by guggulsterone. Long-term dosing of guggulsterone isomers reduces LDL-C levels significantly. In such a way, the pharmacological activity of guggulsterone was performed in the NF-κB pathway. The inhibitory effect of NF-κB signaling in the intestinal epithelial cells (IECs) by guggulsterone isomers has been proved by *in vivo* studies involving murine models as well as cell lines and cultures. Guggulsterone derivatives were specifically designed to have high lipophilicity and low solubility to prolong their time in the intestinal lumen and reduce intestinal absorption to avoid unnecessary NF-κB signal inhibition in other tissues.

## Effect of guggulsterone isomers & derivative on the stages of ulcerative colitis & Crohn's disease

The Z isomer of guggulsterone inhibited acute ulcerative colitis induced by dextran sodium sulfate (DSS) model at a dose of commencing on the day of DSS exposure, once or twice daily by oral gavage (100 mg/kg q.d. and 100 mg/kg b.i.d.) for 7 days. During administration of drug the overall clinical symptoms like behavior, food and water intake, body weight measurements, stool assessment, and the presence of gross blood in stool or anus were monitored. After 7 days, histological and macroscopic examination of colon (disease activity index [DAI] score and measurement of colon length) was done. The administration of Z-GS (100 mg/kg/per day) mitigated clinical symptoms and macroscopic features like inflammation and ulceration of colon, but without statistical significance. On the other hand, Z-GS(100 mg/kg b.i.d.) administration showed significant improvement of clinical symptoms as well as attenuation of macroscopic features associated with acute colitis [9]. These derivatives significantly inhibited the activated NF-κB signals. Kim *et al.* performed a study on mice by oral administration of guggulsterone isomer dose (2000 mg/kg) for two weeks, and had no adverse effect with a dose >2000 mg/kg, or ten times the maximum therapeutic dose. So the guggulsterone isomer also has a protective effect against inflammation with efficacy which highlights its potential for use in a clinical setting for treating IBD [10]. Guggulsterone isomers inhibited NF-κB transcription activity in IEC cells, which were induced by LPS and IL-1β. It did so by inhibiting the phosphorylation of IκBα, which plays a major role in the activation of NF-κB in addition to inhibiting the phosphorylation of IKK and IκB (inhibitor of NF-κB) [9]. The guggulsterone isomer can also inhibit NF-κB even in the presence of the triggering receptor expressed on myeloid cells (TREM-1) agonist (which is known to increase NF-κB activity) and AP-1 activity in RAW264.7 cells, which mimics the inflammatory activity in human intestines [11]. In ulcerative colitis condition, Kim *et al.* conducted a study on experimental mice with DSS-induced colitis, in this study the guggulsterone isomer (200 mg/kg q.d.) showed a significant reduction in the disease severity which was measured using Disease activity index (DAI), colon length and histology. It dramatically reduced both clinical and macroscopic inflammatory indices and resulted in a clear and considerable histological reduction of acute colitis. A similar effect was seen in the therapeutic model as well where GGS (100 mg/kg q.d.) attenuated clinical indices such as body weight change, colon length, and DAI of the mice significantly [10]. Mencarelli *et al.* performed a study on a mouse model, where oxazolone is used to induce the disease. This study report shows that there was a significant reduction in colon inflammation after the administration of (30 mg/kg) of guggulsterone isomers [12] In another study, GGS administered at a dose of (100 mg/kg b.i.d.) to DSS-treated mice significantly diminished the colitis as compared to mice untreated with guggulsterone. It was measured by comparing the DAI, colon length and clinical and microscopical indices [9]. Followed by, in one study conducted to see the effect of guggulsterone on the NF-κB signaling pathway in the epithelial cells of the intestine of both humans and rats (Caco-2 cells and IEC-18), guggulsterone inhibited the NF-κB signaling pathway caused by lipopolysaccharide (LPS) and when pretreated with guggulsterone. This was proved by checking the *ICAM-1* gene (which is triggered by NF-κB) suppression rate and it was around 87% in Caco-2 cells and 73% in IEC-18 cells when pretreated with guggulsterone 1 h back. IL-1β will rise the NF-κB activity. GGS (50 μmmol/l) treated Caco-2 cells and IEC-18 cells over 4 h showed remarkable suppression of NF-κB than untreated cells as detected by using electrophoretic mobility shift assay (EMSA). Moreover, this study also explored the RelA phosphorylation and p38 pathway induced by IL-1β in both cells stimulated by IL-1β. This stimulation will increase the activity of NF-κB. However, GGS treatment did not inhibit them [9]. In another study, the effect of GGS isomers on the NF-κB signaling pathway was studied in the COLO 205 cells and IEC cells stated that guggulsterone isomer inhibited the activity of NF-κB induced by tissue necrosis factor- α (TNF-α) in a dose-dependent way. Guggulsterone isomer blocked IκBα phosphorylation and IKK activity which eventually leads to the inactivation of NF-κB activity. Additionally, the guggulsterone isomer inhibited the mRNA levels of IL-8, an inflammatory cytokine and Monocyte Chemoattractant Protein-1 (MCP-1), a chemokine that influences the macrophage migration during inflammation in a concentration-dependent manner in COLO 205 cells [10]. The guggulsterone isomer was compared with conventional medicine like prednisolone and sulfasalazine, the guggulsterone isomer (200 mg/kg q.d.) showed a remarkable similarity in the efficacy of mitigation of colitis to that of prednisolone(1 mg/kg q.d.) or sulfasalazine (200 mg/kg q.d.) [10]. In 2013, Kang *et al.* performed a study on an IL-10/mouse model of chronic colitis where administration of GG-52 dramatically reduced the severity of colitis as measured by histology and lowered IKK activation in dendritic cells in colonic tissue along with inhibition of NF-κB signaling pathway [13]. In 2016, Zhang *et al.* performed a study to study the effect of GGS on LPS-treated Raw264.7 cells where GGS dramatically reduced the increased mRNA expression of proinflammatory cytokines such as IL-1β, TNF- α, iNOS and NF-κB activity [14].

## Conclusion

This review highlights the strong connection between guggulsterone and its role in modulating intestinal inflammation by interfering with the NF-κB pathway and inflammatory cytokines, which play a vital role in the pathogenesis of IBD. Specific bio compounds which have more efficacy and less toxicity must be identified and more *in vivo* studies as well as clinical trials on this topic need to be carried out. Currently, available treatment options for IBD have limitations such as relapse, high cost and unwanted side effects. Guggulsterone compounds are significantly effective in treating arthritis and hyperlipidemia through various mechanisms. As a result, there is the prospect of producing and testing guggulsterone derivatives as a therapy for IBD.
